# In Depth Breadth Analyses of Human Blockade Responses to Norovirus and Response to Vaccination

**DOI:** 10.3390/v11050392

**Published:** 2019-04-26

**Authors:** Joel Haynes, Virginia Perry, Evelyn Benson, Alisa Meeks, Gayle Watts, Heather Watkins, Ralph Braun

**Affiliations:** Vaccines Discovery Research, Takeda Pharmaceuticals, Cambridge, MA 02139, USA; joelroberthaynes@gmail.com (J.H.); rideskiclog@gmail.com (V.P.); evelyn.benson1@montana.edu (E.B.); alisa.meeks@gmail.com (A.M.); wa7@msn.com (G.W.); heather.watkins@takeda.com (H.W.)

**Keywords:** norovirus, VLP, blockade, vaccine

## Abstract

To evaluate and understand the efficacy of vaccine candidates, supportive immunological measures are needed. Critical attributes for a norovirus vaccine are the strength and breadth of antibody responses against the many different genotypes. In the absence of suitable neutralization assays to test samples from vaccine clinical trials, blockade assays offer a method that can measure functional antibodies specific for many of the different norovirus strains. This paper describes development and optimization of blockade assays for an extended panel of 20 different norovirus strains that can provide robust and reliable data needed for vaccine assessment. The blockade assays were used to test a panel of human clinical samples taken before and after vaccination with the Takeda TAK-214 norovirus vaccine. Great variability was evident in the repertoire of blocking antibody responses prevaccination and postvaccination among individuals. Following vaccination with TAK-214, blocking antibody levels were enhanced across a wide spectrum of different genotypes. The results indicate that adults may have multiple exposures to norovirus and that the magnitude and breadth of the complex preexisting antibody response can be boosted and expanded by vaccination.

## 1. Introduction

Noroviruses are a highly prevalent pathogen associated with approximately 20% of diarrheal disease worldwide and are responsible for greater than 200,000 deaths each year [1,2,3]. All age groups are susceptible to infection; however, the greatest incidence of disease occurs in children under the age of 5 years that can cause up to 70,000 deaths in children in developing countries. In healthy adults, the disease is usually self-limiting and only rarely becomes problematic. However, in the immunocompromised (all ages) and in the elderly with underlying health problems and immunosenescence, mortality becomes higher [2,4]. In addition to the effect on individual health, the worldwide economic burden of norovirus disease is estimated to be approximately 60 billion dollars per year [5]. As the understanding of the prevalence and burden of norovirus disease strengthens, the need for preventative and therapeutic measures becomes more apparent, including the development of an effective vaccine [6,7].

One of the interesting features of norovirus disease, but one that is difficult to address for vaccine development, is the large number of different genotypes, and the multiple strains within each genotype, that circulate in the human population [6,8]. There are three genogroups of norovirus that can infect humans, which include Genogroups I, II, and IV, although the GI and GII viruses are the major causes of human disease. Within each of the GI and GII genogroups there are many genotypes, of which the GII.4 genotype noroviruses are of particular importance because of their higher incidence and their association with more severe disease [8,9]. The GII.4 noroviruses are unique in their continuous evolution and cyclic emergence of new pandemic strains that may avoid the existing immunity in the population [10,11]. For a norovirus vaccine to be highly efficacious it must be able to protect against the GII.4 strains in circulation and ideally, newly emerged GII.4 strains. The other norovirus genotypes appear to have a more stable evolution and show few changes in sequence over time, although they do remain in circulation and contribute to a significant portion of the norovirus infections [8,12]. Non-GII.4 noroviruses may be responsible for additional outbreaks, and the specific genotypes responsible can vary. A major challenge in norovirus vaccine development is to prepare formulations to elicit cross-reactive antibody responses capable of broad neutralization of multiple genotypes.

A major roadblock in norovirus research has been the lack of a suitable culture system that would allow in vitro growth and replication of the virus. Because of this, two common vaccine platforms—attenuated or killed viral vaccines—have not been available for the development of norovirus vaccines. The discovery of virus-like particles (VLPs) for norovirus and their application to the area has been critical in advancing the understanding of norovirus disease and the development of methodologies to characterize immune responses [13]. VLPs are generated by the expression of the viral capsid protein VP1 in cell culture, which can self-assemble to structures that mimic the virus particles. VLPs are believed to reproduce many features of the virus and have been used to study the structure and function of norovirus. Norovirus VLPs have also been shown to be antigenic and most vaccine candidates are based on different variations of VLPs [6,7,14,15,16,17,18]. Candidate vaccines have been composed of purified VLPs or P-particles that express the protruding domain of the VP1 protein. Other vaccines have employed vectors that express VP1 proteins that would generate VLPs after delivery of the vaccine. Multivalent formulations of VLPs or chimeric VLPs have also been tested to address the breadth of coverage needed for a norovirus vaccine. Takeda Vaccines has been developing a bivalent VLP-based vaccine against norovirus that includes one VLP representing each of the major genogroups. The genogroup I VLP is based on the Norwalk virus—the first identified norovirus—and is genotype GI.1. The second VLP is a “consensus” GII.4 VLP made by combining sequences from the Houston (2002), Yerseke (2006) and DenHaag (2006) GII.4 strains [19,20].

To evaluate vaccine efficacy, measurement of immune responses to the vaccine is needed with preference for functional assays that may better relate to efficacy than an assay based solely on antibody binding [21]. For vaccines against viruses, neutralization assays are most commonly used as the functional assay for measurement of antibodies that stop or neutralize viruses from infecting cells. Norovirus infection systems have only recently become available [22,23], but these infection models are still highly variable and are not yet suitable to establish neutralization assays with the reproducibility and reliability needed for the routine sample testing, although they have been valuable to characterize neutralizing human monoclonal antibodies [24]. Blockade assays have been an extremely valuable surrogate for virus neutralization assays and these assays use norovirus VLPs as substitutes for whole virus, and measure the ability of serum antibodies to block the binding of the VLPs to cell surface carbohydrates [25,26]. Noroviruses interact with cell surface carbohydrates, such as the Histo-Blood Group Antigens (HBGAs) that have been identified as important attachment factors for norovirus [27,28]. The ability of antibodies to block attachment of VLPs is considered a functional activity, and blocking antibodies have been found to be a correlate with protection in human infection and challenge models [29,30,31,32]. To measure blockade antibodies, synthetic HBGAs and salivary mucins can be used as the carbohydrate substrates. However, these reagents have considerable variability and only a limited range of VLPs show binding to each type. The use of pig gastric mucin (PGM), which contains several human HBGAs, as the substrate for VLP binding has been invaluable in enhancing reproducibility and the range of VLPs that bind [31], and PGM is the substrate of choice for the blockade assays described in this paper.

The Takeda TAK-214 norovirus vaccine has been found to be safe and immunogenic in several human clinical trials [20,33,34,35]. To gain a better understanding of the capabilities of the vaccine, the magnitude and breadth of blocking antibodies that are elicited, or boosted, by the vaccine need to be characterized. This requires many different VLP blockade assays because of the large number of norovirus genotypes that circulate. Since each VLP has its own unique characteristics, a rigorous optimization is needed to develop reliable assays for each VLP. The work described here illustrates several parameters that were key to the optimization of blockade assays for 20 different norovirus VLPs. Screening of adult human sera using the established blockade assays showed a complex repertoire of blocking responses, before and in response to vaccination. The blockade assays optimized for a large VLP panel will be important to delineate the immune responses to noroviruses, and to help identify those responses that may be protective against disease.

## 2. Materials and Methods

### 2.1. Assay Buffers

Phosphate buffers (100 mM) with different pH values were prepared by mixing ratios of the stock 100 mM monobasic (NaH_2_HPO_4_, Thermo Fisher Scientific, Waltham, MA, USA) and dibasic (Na_2_HPO_4_, Thermo Fisher Scientific, Waltham, MA, USA) solutions, to generate the desired pH. If needed NaCl (Sigma-Aldrich, Natick USA) was then added to 150 mM from a 5 M stock and Tween 20 (Thermo Fisher Scientific, Waltham, MA, USA) added to 0.05%. The final pH was adjusted by adding either the monobasic or dibasic stock.

### 2.2. Production of VLPs

VP1 amino acid sequences obtained from GenBank were used to commercially synthesize a mammalian codon optimized nucleotide gene sequence for each particular VP1 protein (ATUM, Newark USA). The GenBank Accession numbers for all VLPs are listed in Table A1. Restriction sites were engineered onto the ends of the synthetic genes to facilitate cloning into the AdEasy Adenoviral Vector System Cloning kit from Agilent that was used to produce the recombinant adenovirus clones. The recombinant adenoviruses were used to infect Vero cells at a multiplicity of infection of 300, and cultures were harvested after 4 days. The supernatant from cells was removed and the adherent cells were treated with a PBS and 0.1% Tween solution for 5 min with rocking at room temperature to lyse cells. The lysate was clarified by centrifugation (400× *g*) and filtered through a 0.45 µM syringe filter and then spun into a 40% sucrose cushion in an ultracentrifuge (100,000× *g*) as described elsewhere [36]. Purity of VLPs was assessed by SDS-PAGE and concentration determined by BCA assay. The VLPs were frozen at −80 °C in the 40% sucrose/PBS buffer and used in blockade assays by dilution into assay buffer. Once thawed, VLPs were stored at 4 °C for up to one year or until loss of PGM binding was evident. The vaccine antigen VLPs GI.1 and GII.4 CN are the only two VLPs not made by expression in mammalian cells. These VLPs were obtained from manufacturing runs of vaccine VLPs that are produced in insect cells using a baculovirus expression system.

### 2.3. Detection Antibodies

VLP specific antibodies were generated by injecting rabbits with purified VLPs using Gerbu Adjuvants^®^ (GERBU Biochemicals GmbH, Gaiberg, Germany) to generate hyperimmune serum. Sera from 3 rabbits were pooled and the IgG fraction was purified. The purified IgG was used as the detection antibody in the PGM binding and blockade assays. Table A1 lists the different detection sera used and working concentrations for VLPs and antibodies for the different assays.

### 2.4. Human Serum Samples

Unvaccinated human sera (individual or pooled) were obtained from several commercial sources (Sigma-Aldrich, Natick USA; BioIV, Westbury USA; Innovative Research, Novi USA; Jackson ImmunoResearch, West Grove USA). These adult human sera were evaluated for blocking activity against the panel of VLPs to identify those with positive blockade responses for optimization experiments.

Clinical trial samples were obtained under written informed patient consent from two clinical trials NOR-201 (NCT02142504; IRB00000231 Cincinnati (FWA00002988), IRB00000773 San Diego (FWA00006704), IRB00003226 San Francisco (FWA00008684) Coral Gables (FWA00018376) Rochester (FWA00001317) Austin (FWA00008684), IRB00000158 St. Louis (FWA0005304), IRB00000533 Rochester (FWA00009386), IRB00000077 Houston (FWA00000286)) and NOR-210 (NCT02475278; IRB00003226 Austin (FWA00008684)). Samples were selected from individuals who were given the selected adult dose for TAK-214; a vaccine dose of 15 µg GI.1, 50 µg GII.4CN, and 500 µg Aluminum hydroxide (Al(OH_3_). Subjects in the NOR-201 trial were administered a vaccine that contained 50 µg Monophosphoryl Lipid A (MPL) per dose in addition to the VLPs and Al(OH_3_) amounts noted above. Samples from both trials included pre-bleeds and samples taken approximately 30 days after a single dose of vaccine. Both trials were conducted in subjects 18 to 49 years of age in North America. Samples from 25 subjects were obtained from participants in the NOR-201 trial conducted in 2014. Samples from 24 subjects were obtained from participants in the NOR-210 trial conducted in 2015.

### 2.5. Pig Gastric Mucin (PGM) Coated Plates

NUNC 96-well plate flat bottom Maxisorp plates (Thermo Fisher Scientific, Waltham, MA, USA) were coated with 100 µL of a 5 µg/mL solution of PGM (Sigma-Aldrich, Natick, MA, USA) in PBS (Thermo Fisher Scientific, Waltham, MA, USA) overnight at 4 °C, or 1 h at 37 °C. Following coating, plates were washed 3 times with 300 µL/well PBS and 0.05% Tween 20 (PBST), and then blocked with 200 µL/well of StartingBlock (PBS) Blocking Buffer (Thermo Fisher Scientific, Waltham, MA, USA) for 1 h at room temperature. Plates were washed 3 times with PBST before use.

PGM contains several human HBGAs that are bound by VLPs, but does not contain the Blood Group B antigen which is recognized by GII.12 noroviruses, and GII.12 VLPs do not bind PGM coated plates [30,37,38]. Blood group H antigen found in PGM can be converted to B antigen using the enzyme, B antigen glycosyl transferase (GTB). Conditions were developed in which the blocked PGM coated plates were treated with recombinant human blood group B transferase/GTB Protein (R&D Systems, Minneapolis, MN, USA) to generate B antigen (manuscript in preparation). For this, blocked PGM coated plates were incubated with 0.25 µg/mL GTB protein in assay buffer (50 mM HEPES pH 6.5, 1.5 mM UDP-galactose, 5 mM MnCl_2_) for 6 h at 37 °C. Plates were washed 3 times and 150 µL PBST was added to each well and held overnight at 4 °C before use. The GII.12 (2010) VLP binds to PGM plates treated with GTB, and these GTB treated PGM plates are used for the GII.12 VLP blockade assay.

### 2.6. Immunological Assays

The PGM binding assay was performed by adding VLPs to blocked PGM-coated plates and incubating for 1 h at 22 °C or 37 °C. Following 3 washes with PBST, detection antibody specific for the VLP diluted in assay buffer was added and the plates were incubated at room temperature for 1 h and then washed 3 times with PBST. A goat anti-rabbit IgG-HRP (Southern Biotech, Birmingham, AL, USA; #4030-05) secondary antibody was then added at 1:3000 dilution in assay buffer and incubated for a further 1 h at room temperature. Following 3 washes, enzyme substrate (ABTS Peroxidase Substrate, KPL) was added and allowed to react for 12 min at room temperature. ABTS Peroxidase Stop Solution (KPL) was then added and plates were read at a wavelength of 450 nM in a Molecular Devices plate reader using SoftMax Pro Software (Molecular Devices, Downingtown, PA, USA) to obtain the Optical Density (OD) of each well.

For blockade assays performed on the clinical samples, samples were first diluted 1:15 in assay buffer and added to 96-well dilution plates (NUNC, conical bottom non-stick plates) and serially diluted 4-fold down the plate. A positive serum control and a VLP-only control were run in columns 11 and 12 respectively. Following the dilution step, VLP diluted to 2× in assay buffer was added to the dilution plate in 100 µL bringing the total volume in each well to 200 µL. The plates were sealed and incubated for 18 h in an environmental chamber set at 22 °C. The following day 150 µL of the solution from the dilution plate was transferred to the PGM coated plate and the remaining steps followed those of the PGM binding assay described above. The OD data was curve fit using the Log-Logit function in SoftMax pro. Blocking titers were calculated as the serum dilution interpolated at ½ the maximum OD for that plate and represents the serum dilution that produces a 50% reduction in VLP binding to PGM. The maximum OD for the plate was calculated from the VLP only column. The background of the assay was assigned a titer of 30 which is the starting dilution of the sera. Any samples with titers below 30 were given a value of <30 and assigned a value of 15 for performing calculations. Statistical analyses of the data were performed using Microsoft Excel and GraphPad Prism (GraphPad, San Diego, CA, USA).

For testing clinical samples, to reduce variability, all samples were tested on the same day, by the same operator for each individual blockade assay (10 plates total). The pre- and postvaccination samples were run on the same plate, to also reduce possible variability. To conserve serum and support the many assays performed on each sample, and because of the low variability found between duplicate curves in the optimized assays, samples were run as single dilution curves. Positive controls, also run as single dilution curves, were tested on each plate during the sample testing and were used to monitor the assays. The overall variability in positive controls values measured during the testing was approximately 10% CV (standard deviation/mean − 100) indicating that the between assay variability was low.

#### 2.6.1. Optimization of PGM Binding

A screening procedure was developed to identify conditions for optimal binding of each VLP to PGM following an overnight incubation in buffer to mimic the conditions used for the blockade assays. The 96-well dilution plates were set up with 4 wells to test each condition and the ODs measured in the 4 replicate wells were averaged to compare conditions. VLPs were incubated in 200 µL of phosphate assay buffers of different pH (pH 6.5, 7.0, and 7.5) with and without 5% skim milk for 18 h at 22 °C without antibody. The following morning, 150 µL of the solutions were transferred to the PGM coated plates and the assay completed as described above. The OD values indicated the strength and overnight stability of VLP binding to PGM.

#### 2.6.2. Optimization of Blockade Assays

Once buffer conditions were evaluated for each VLP, a final set of experiments were performed to optimize the assay conditions to maximize the sensitivity for measuring blocking antibodies. Commercial human sera (negative and 3 positives with a range of strengths for each VLP blockade assay) were screened under different conditions: buffer pH (6.5 and 7.5), primary incubation (18 h and 1 h), and PGM binding temperature (22 °C and 37 °C). For each serum and condition combination, a titer was calculated. If low binding of the VLP to PGM occurred under any of the conditions (less than half of the optimal binding OD) that condition was designated as “failed” and titers were not calculated. This ensured that the titers were calculated from binding curves with sufficient magnitude and reliable curves. For each of the 3 positive sera, titers were used to generate a ratio of titers measured under the two conditions being compared to assess which condition generated the highest titers.

## 3. Results

### 3.1. Optimal Conditions for Blockade Panel

Previous development of a GII.4 (2012) Sydney blockade assay in our laboratory had identified several parameters that greatly improved the measurement of blocking antibodies. The conditions with most impact were an overnight incubation of VLP with antibody at 22 °C, conducting the PGM binding incubation at 37 °C, and the use of VLPs expressed in mammalian cells. For this work, the buffer was PBS (pH 7.4) with Tween 20 (0.5%) and 5% skim milk.

As more VLPs were generated for use in the blockade assay panel, several VLPs were found not to work well in the conditions used for the GII.4 (2012) assay. A more comprehensive evaluation of optimal assay conditions was therefore required for the extended VLP panel.

#### 3.1.1. Buffer Evaluation

The PGM binding activity of VLPs was addressed with a screening procedure that evaluated buffer pH (6.5, 7.0, and 7.5), the presence or absence of 5% skim milk and incubation temperature for PGM binding (22 °C or 37 °C) as described in the Materials and Methods. The average OD measured in 4 replicate wells was used to calculate the relative strength of binding under the different conditions. The strongest binding (highest OD) was assigned a value of 100%. Table 1 lists the results across a panel of different VLPs with shading in red representing the strongest binding and in green the weakest binding. 

Several general characteristics of VLP binding are apparent in Table 1 and these were used to identify conditions of most importance to guide the optimizing of the blockade assays themselves.

The presence of 5% skim milk reduced the binding of all VLPs under all conditions, with the only exception being the binding of GII.4 CN at pH 7.5 and incubation at 37 °C. In some cases, the presence of skim milk very strongly inhibits binding (see GI.4 VLP). Skim milk is commonly used in plate-based assays to reduce nonspecific binding, but it is a complex uncharacterized component that can ideally be removed from buffers when assays advance towards routine testing. Glycans found in human milk have been found to bind to norovirus VLPs and block association with HBGAs [39], thus inhibitory glycans may also be present in skim milk products. Based on the buffer investigations, the 5% skim milk was removed as a buffer component since it selectively influences binding of VLPs to PGM. To reduce nonspecific binding to the plates in the absence of skim milk, a commercial blocker was used to block the PGM-coated plates prior to their use.

In Table 1, there is also a noticeable trend for the strongest binding for the majority of the VLPs to occur at a pH of 6.5, although several VLPs bound well across the range of assay buffer pHs. Notably, all the VLPs that had poor binding at pH 7.5 showed improved binding at pH 6.5 (e.g., GI.5 and GII.4 CN). This finding was helpful in addressing the inconsistent binding behavior of several VLPs in the original pH 7.4 buffers. Problems with variability in blockade assays were found to be most often resolved by lowering the assay buffer to pH 6.5. Because the pH 7.0 buffer does not appear to offer any additional advantage over the binding found in either the pH 6.5 or 7.5 buffers, it was not included in the subsequent optimization of the blockade assays.

One final trend of interest was that binding to PGM at 37 °C generally produced the highest binding with typically a 10–20% improvement over binding at 22 °C. However, for some VLPs under nonoptimal buffer conditions there was actually a reduction in binding at 37 °C (see GI.5, GII.4 CN, and GII.17 1978). This apparent sensitivity to temperature is not related to the stability of the VLPs, but rather appears to be related to the strength of the VLP interaction with PGM. Two of the VLPs (GI.6 and GIV.1) were found to show strongest PGM binding at 22 °C irrespective of the buffer, and therefore the PGM binding step in blockade assays with these VLPs are performed at 22 °C (see below).

#### 3.1.2. Blockade Assay Optimization

Based on the results of the assay buffer evaluation, the optimization of VLP blockade assays was carried out by comparing test results obtained under different conditions of primary incubation (18 h and 1 h), PGM binding temperature (22 °C and 37 °C) and assay buffer pH (6.5 and 7.5). This final optimization was needed to identify conditions that measured the blocking titers with the most sensitivity and reliability, rather than just selecting best conditions based on VLP binding, since stronger binding to PGM could actually reduce sensitivity of the blockade assays. For these investigations commercially obtained human sera were used. Unvaccinated human serum has a mixture of anti-norovirus antibodies from past exposures that should also contain cross-reactive antibody populations, which are an important population for the blockade assay panel to capture.

Human sera (individual and pooled) were screened to identify sera that were negative, or with high, medium and low titer for each of the VLP blockade assays. As described in the Material Methods section, the sera were then tested under a variety of conditions and the corresponding titers were used to identify the preferred conditions for measuring blocking activity. Table 2 summarizes these investigations and lists the outcomes and the final conditions selected for each of the assays. The data in Table 2 is a summary of 640 individual tests and illustrates the considerable effort undertaken to verify and optimize these assays. The ratios listed in Table 2 are used as an indication of the best conditions to obtain the most sensitive measurement of blockade titers. Ratios over 1 indicate the numerator condition is the optimal condition. Cells that list either “37°C Fail” or “pH 7.5 Fail” are situations where the VLP binding to the PGM was below a predetermined minimum value and these data were not used in the calculations. The final two columns in the table list the assay conditions selected for each of the different blockade assays. 

For the primary incubation all calculated ratios were above 1, indicating that the 18h incubation in general was the best condition for all blockade assays. In no case was the titer measured following 18 h incubation lower than using 1h incubation, although usually the titers were the same under these conditions, especially with higher titer sera. The 18 h incubation does not raise the assay background and did not convert any of the negative responses to positive, indicating that the enhancement is specific. In past experiments, some sera and monoclonal antibodies have been found to be greatly influenced by the longer incubation time and titers up to 10-fold higher are generated under the longer incubation time. For this reason, the 18 h incubation is preferred as it may best capture the binding of all different antibody populations. The 18 h incubation may allow binding to hidden epitopes as found for West Nile Virus neutralization studies [40] where “breathing” of the particle may allow antibodies to interact with the occluded sites. Using a higher temperature for the primary incubation may also have a similar effect [10,41], however, the 18 h incubation was chosen as it is logistically simpler to divide the lengthy blockade assays over two days especially when large numbers of samples are being tested. Using an extended time for the primary incubation has also been used in qualified assays that have been used to measure responses for human vaccine clinical trials [42,43]. 

For the PGM incubation step listed in Table 2, GI.6 and GIV.1 assays are listed as failing at higher temperature which is a result of their poor binding to PGM at 37 °C. Under these conditions titers were not calculated since proper binding curves could not be generated from the low OD values. These two assays are therefore performed at 22 °C since they are unreliable at higher temperature. For VLPs that bind well to PGM at 37 °C, the PGM incubation step in the blockade assays all had ratios above 1 indicating that the higher temperature slightly increased sensitivity and higher titers were obtained. 

The third condition to be evaluated and summarized in Table 2 was the assay buffer pH. Although binding at pH 6.5 appeared to be a reasonable condition for all VLPs, the data indicate that for measuring titers, this may not be the optimal pH. This is especially apparent for the GII.17 and GII.4 strains that have ratios less than 1. The GII.17 blockade assays, for example, have ratios near 0.5 indicating that titers measured at pH 7.5 are about twice those measured at pH 6.5. In general, pH 7.5 may be most sensitive for measuring blocking titers, however, the large number of VLPs that have unreliable binding at that pH (listed as pH 7.5 fail) must be tested at pH 6.5 to generate consistent outcomes. 

Although no universal assay conditions could be found that worked for all assays, a set of 3 assays have been established; pH 6.5 buffer with 37 °C PGM binding, pH 6.5 buffer with 22 °C PGM binding, and pH 7.5 buffer with 37 °C PGM binding step. All other conditions for the assays are the same.

### 3.2. Testing of Samples from Individuals Vaccinated in Clinical Trials 

The final optimized conditions for each of the blockade assays were used to analyze samples selected from two Takeda clinical trials for comparison of antibody responses in subjects pre- and postvaccination, and the influence of inclusion of the MPL adjuvant on the response to vaccine.

Table A2 and Table A3 in the appendix list the blockade titers measured in serum from subjects before and after vaccination with the TAK-214 vaccine. Samples selected from the NOR-201 study were from subjects who received the vaccine formulated with MPL, while all the samples from the NOR-210 study were from subjects vaccinated with a formulation that did not include MPL. Assessing the effects of vaccination in a primed population with different exposure histories is not simple and several different approaches have therefore been used to help fully describe the dataset.

As an initial assessment of the blockade data, the percent of responses above the assay background of 30 (Table 3) and the geometric mean titers (GMTs) (Table 4) were tallied and compared across the assay panel. Table 3 lists the percentage of individuals with blockade responses above background for each of the VLPs pre- and postvaccination. Data are listed separately for each trial and for both trials combined. These measures give an indication of the breadth of the responses, but not necessarily the magnitude. Table 4 lists the calculated GMTs in the same arrangement as Table 3, and is an indicator of the magnitude of the response across the different populations.

#### 3.2.1. Preexisting Responses

The breadth and magnitude of the preexisting responses would be influenced by the genotypes that had circulated in these populations prior to the trials. The higher percentage of positives and higher GMTs for the GII.4 strains in both NOR-201 and NOR-210 clinical trials agrees with the prevalence of this genotype. As listed in Table 3 and Table 4, the NOR-201 and NOR-211 trials had similar patterns of preexisting immune responses. Both trials were run in the United States, in the same age group, and within two years of each other, so large differences were not expected. The only difference of note is the consistent lower number and magnitude of preexisting responses to GII.4 in NOR-210. This difference may be due to the sporadic nature of norovirus outbreaks where differences may be expected from one location to another. There were 10 clinical sites in NOR-201 but only a single site in NOR-210.

Within the preexisting measured responses, some reactivity was found to all the VLPs tested. This likely reflects both the response to infection by specific norovirus strains of which a high number remain in circulation, and cross-reactive responses between the strains. Among the individual subjects in the trials, there was great variability in the number of positive preexisting responses. One individual had no positive responses, and another was positive to all 13 genotypes tested (Table A2 and Table A3), and the average number of positive preexisting responses per subject was 6.

#### 3.2.2 Postvaccination Responses

Evaluation of percent postvaccination positive responses showed that 90% and 100% of individuals were positive to the vaccine antigens GI.1 and GII.4 CN, respectively. This is as expected, as TAK-214 generates strong immune responses to the vaccine antigens. Additionally, since GII.4 CN is a mixture of sequences from the GII.4 2002, 2006a, and 2006b strains, these VLPs are also expected to show enhanced blocking antibodies postvaccination. There was also a general boost of positive responses and GMTs to all the VLP genotypes. This will be explored in more detail in the following sections; however, these data indicate that vaccination can boost responses against genotypes outside of the vaccine strains. This general boosting of the breadth is also reflected in the number of positive responses per individual with the average now climbing to 9. 

#### 3.2.3 Role of MPL

One goal of this work was to look further into the possible role of MPL for enhancing the activity of TAK-214. Previous work had found that there was no evidence that MPL enhanced the magnitude of immune responses when it was included in the vaccine formulation, and this work was intended to see if any impact on the breadth of responses was apparent using MPL. All samples tested from the NOR-201 trial had been from individuals given vaccine containing MPL, whereas subjects in the NOR-210 only received vaccine without MPL. Comparing the results between these trials (Table 3, Table 4 and Table 5) indicates that no real differences existed in the responses to vaccines with, or without, MPL. Averaging outcomes across the assays showed that the percentage of positive individuals was the same (79%) in both trials after vaccination, and the GMT and fold-rises were actually slightly higher in the NOR-210 trial (628 versus 467, and 5.7 versus 4.2, respectively). These results indicate that the magnitude and breadth of responses did not differ significantly when MPL was included in the vaccine formulation.

#### 3.2.4 Geometric Mean Fold-Rise of Blockade Titers

Another method to evaluate the effect of vaccination is to examine the geometric mean fold rise (GMFR) in blockade titers. Table 5 lists the fold increases measured for blocking antibodies against the different VLP strains. The two trials are once again shown separately and in combination.

**Figure 1:**
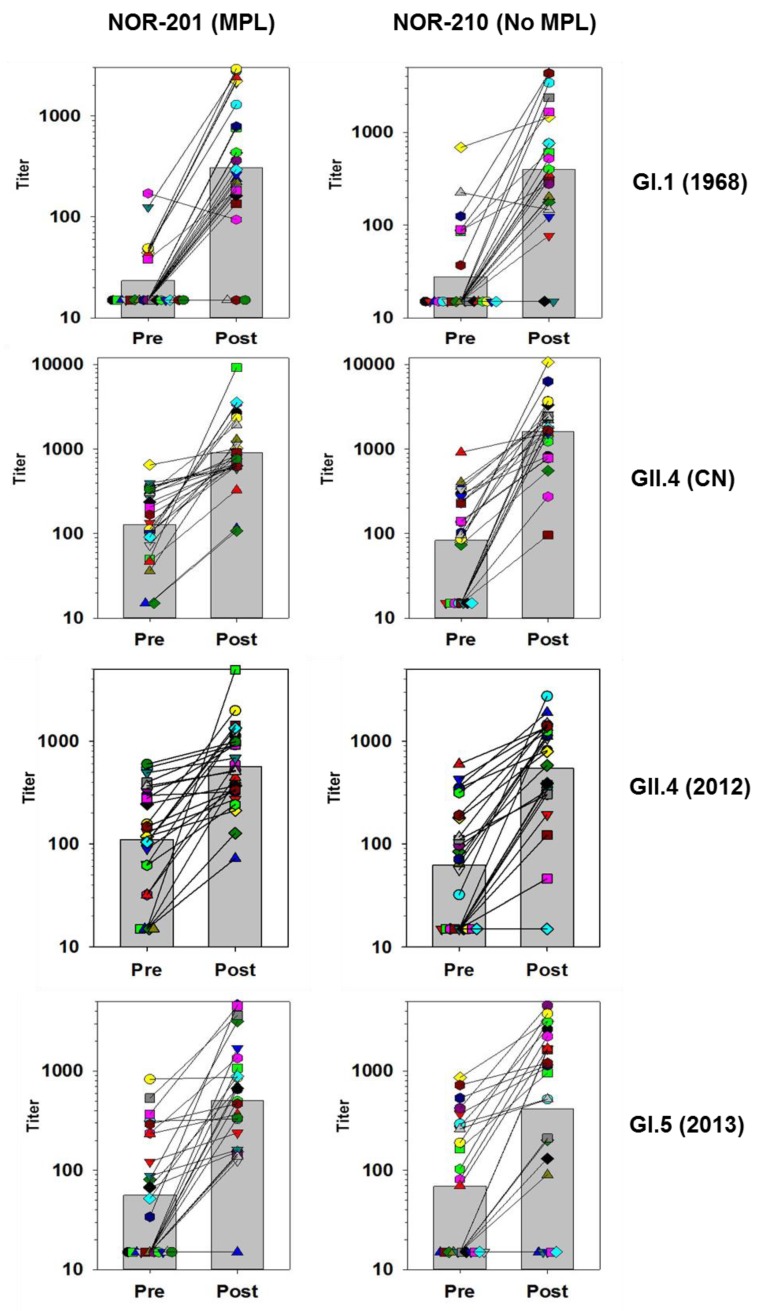
Blockade titers specific for GI.1 (1968), GII.4 (CN), GII.4 (2012), and GI.5 (2013) measured in individual sera before and after vaccination with TAK-214 in the NOR-201 and NOR-210 clinical trials. The subjects tested from NOR-201 were given vaccine that contained MPL adjuvant and subjects in NOR-210 received vaccine without MPL. Individual subjects are different colors and matched sera results are connected by solid lines. The bars in the background represent the geometric mean titer of the subjects. Samples below the assay lower limit of quantitation of 30 are assigned a value of 15.

**Figure 2:**
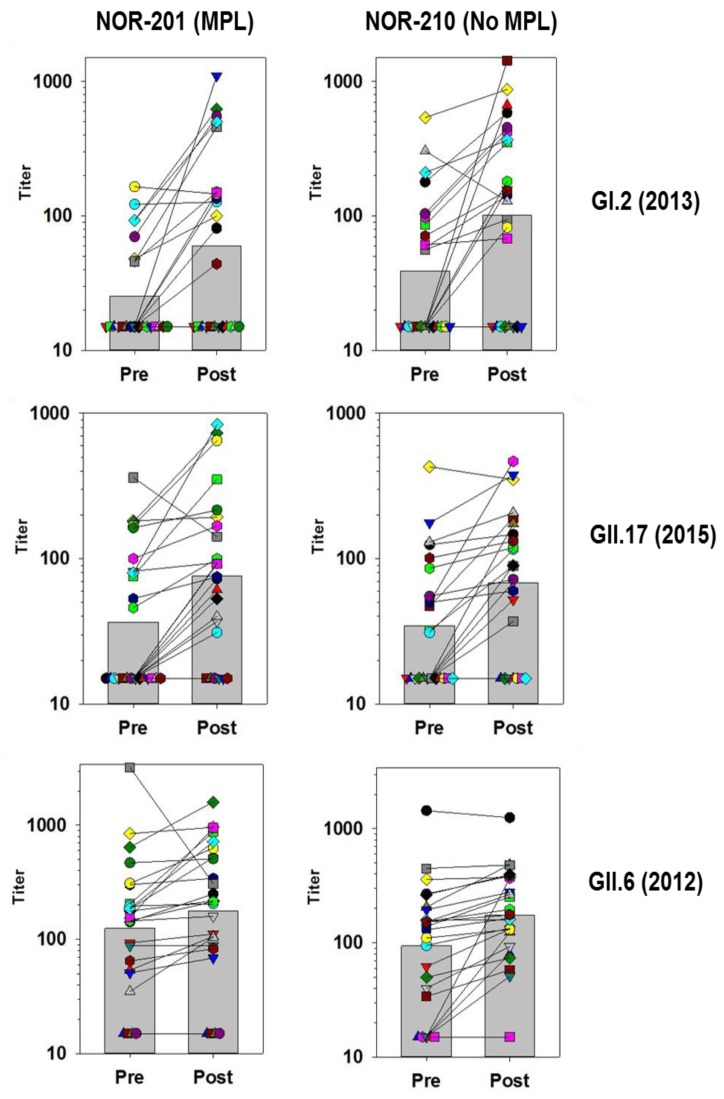
Blockade Titers Specific for GI.2 (2013), GII.17 (2015), and GII.6 (2012) measured in individual sera before and after vaccination with TAK-214 in the NOR-201 and NOR-210 clinical trials. The subjects tested from NOR-201were given vaccine that contained MPL adjuvant and subjects in NOR-210 received vaccine without MPL. Individual subjects are different colors and matched sera results are connected by solid lines. The bars in the background represent the geometric mean titer of the subjects. Samples below the assay lower limit of quantitation of 30 are assigned a value of 15.

In general, vaccination increased antibodies to all antigens tested. Vaccination elicited a greater than 4-fold increase in GMFR against the vaccine antigens (GI.1 and GII.4 CN), as well as nonvaccine antigens such as GI.5, GI.6, GII.4 (2009), and GII.4 (2012). Although the magnitude of the rise in antibodies was comparable for several assays, the character of the boosting among the individual subjects differed. To better illustrate these differences, Figure 1 and Figure 2 below track the individual subjects and their responses before and after vaccination for several of the VLPs. The responses have been categorized into those with high GMFRs and those with low GMFRs.

### 3.3. High GMFR Responses

VLPs with blockade responses that displayed high GMFRs showed a broad boosting of responses across virtually all individuals irrespective of their preexisting responses, and a strong increase in the overall GMT of the population (Figure 1).

The Norwalk virus (GI.1) was the first strain of norovirus discovered and still circulates to the present day, although at a low level, and this is reflected in the low number of individuals categorized as having a positive response before vaccination. After vaccination, most individuals did show positive responses indicating a strong vaccine-antigen specific response as found previously in human trials.

In contrast to the Norwalk strain, the GII.4 strains of norovirus are the most common currently in circulation. Therefore, as expected, the number of individuals with positive preexisting responses was much higher for GII.4 CN than to GI.1. Figure 1 also shows that all individuals responded to vaccination against the vaccine GII.4 antigen. The larger fold-rise measured in the NOR-210 samples was mainly a result of the lower preexisting titers in this group as noted previously.

One critical feature needed for a norovirus vaccine is the coverage of immune response across the various strains of the GII.4 genotype because of their predominance in causing norovirus disease. The GII.4 (2012) Sydney strain emerged after the GII.4 CN antigen was constructed. The GII.4 Sydney blockade responses showed a strong and consistent boost across the individuals postvaccination. This indicates that the GII.4 CN antigen can elicit or boost immune responses specific for newly emerged GII.4 strains. Additionally, all the GII.4 strains tested, showed GMFRs above 4 (Table 5) confirming the strong coverage of the GII.4 strains by vaccination with the TAK-214 vaccine.

The strong blockade responses against GI.5 and GI.6 were significant as these genotypes are not represented in the vaccine. As with other responses that had a strong GMFR, the GI.5 blocking antibodies showed a clear majority of individuals with a boost following vaccination, even those that had negative prevaccination titers.

### 3.4. Medium and Low GMFR Responses

Many of the blockade responses to VLPs had GMFRs below 4-fold (Table 5). In comparison with the high GMFR responses the trend for responses against these VLPs was that fewer individuals responded to vaccination, there was no consistent boosting across the population and different patterns of responsiveness were apparent against the different VLPs. Individuals could show considerable boosting of both positive and negative preexisting responses, while other individuals did not respond. Further, a preexisting response did not necessarily guarantee boosting postvaccination (Figure 2).

GI.2-specific responses were not common prior to vaccination but boosting of some individuals was found. Boosting was not consistent across the subjects, some individuals showed very strong responses to vaccine, while others did not. Boosting was not necessarily dictated by preexisting responses since some preexisting positive responses did not boost, while subjects considered negative could boost well.

The GMFR measured for GII.17 (2015) VLPs was similar to GI.2, but the pattern differed slightly. For GII.17 (2015) responses, the fold rises were not as high among individuals, but more individuals responded. As a result, more subjects had measurable levels of blocking antibodies to GII.17 (2015) than GI.2.

The pattern of responses to GII.6 shown in Figure 2 differ from the GI.2 and GII.17 (2015) responses. GII.6 blocking antibodies were common prior to vaccination, and although the levels increased they only rose slightly postvaccination, with a few individuals showed very strong boosting. The large decrease in blocking titers for the paired sample (grey squares) in the NOR-201 trial might be explained by an infection with a GII.6 strain just prior to the first sampling. Although the GMFR calculated for GII.6 response was low, the postvaccination titers indicate that most individuals had a positive level of antibodies to GII.6, and for this situation the GMFR alone does not fully reflect the GII.6 antibody response.

## 4. Discussion

An important characteristic of a broadly efficacious norovirus vaccine will be the ability of the vaccine to generate, or boost, responses against the numerous norovirus strains and genotypes that exist. These studies can shed light into the potential of the vaccine to protect against norovirus genotypes that are in circulation, and to newly emerging strains; especially those of the GII.4 genotype. Previous testing of samples from clinical studies of the Takeda norovirus vaccine candidate TAK-214 have found strong responses against vaccine antigens and, in addition, evidence for cross-reactivity against nonvaccine genotypes and strains [20,33,35]. We have sought to extend these investigations with a more comprehensive evaluation of the breadth of blocking antibody responses.

The VLPs selected for testing were meant to represent the most recent nonvaccine strains from as many representative genotypes as possible. It would be impossible to capture information against all the norovirus genotypes because several VLP types do not bind PGM, and full-length VP1 sequences are missing for several genotypes. However, the panel used for testing here is the largest to date that captures blocking activity against a wide range of different genotypes and strains. The panel has many strains within the GII.4 genotype because of the historical importance of the GII.4 viruses in causing disease and the ability to evolve into new strains. For the other genotypes, Parra et al. [12] suggest that their evolution may be static with only small changes in the sequences over time. Thus, having only a single strain of VLP from these genotypes should be representative of the genotype. For this reason, we feel that the coverage that the panel of VLPs provides will be a reasonable assessment of the breadth of blockade responses to norovirus.

With the exception of the two vaccine antigens, which were produced in insect cells, the VLPs chosen for the panel were expressed in mammalian cells using an adenovirus vector system. Lindesmith et al. [41] have found that there were differences in the binding of antibodies to VLPs expressed from mammalian and insect cells, even though the sequences were the same. The difference in binding was thought to be the result of a higher flexibility of the mammalian expressed VLPs. A more flexible VLP may expose hidden epitopes and potentially allow more antibody populations to bind than to VLPs expressed in insect cells. We have also found differences in binding with two different strains of VLPs (GI.3 and GII.4 2012) and side by side comparisons found higher blocking titers to the mammalian-expressed VLPs. Given the possibility that assays with mammalian VLPs may be able to detect additional antibody populations we have used mammalian-expressed VLPs exclusively for all nonvaccine VLPs in our panel.

As new VLPs were generated and tested, several were found to have erratic behavior under what had been standard conditions. An extensive investigation of buffer and assay conditions for each VLP was performed to ensure that all blockade assays were optimized. An antibody response generated from vaccination or infection would contain many antibody populations that recognize the multiple epitopes of the immunizing or infecting strain. However, against a different strain, with a different amino acid sequence, only a fraction of the antibody response would be expected to cross-react because of the absence of some epitopes, and sequence differences of other epitopes that would reduce the strength of binding. These cross-reactive antibodies would be expected to contribute to protection against diverse strains. Antibody responses generated by Cervarix®—a VLP-based vaccine against Human Papilloma Virus (HPV)—were found to be 100 to 1000 times lower against nonvaccine strains than against vaccine antigens even though protection was found against the nonvaccine strains [44].

With the panel of blockade assays available, samples from two vaccine clinical trials were tested to determine the breadth and strength of responses before and after vaccination, and the potential influence of MPL. Differences were observed in the levels of preexisting blocking antibody among individuals, indicating a range of exposure histories. Following vaccination, variability was also found in the strength and breadth of responses of individuals to vaccination. Further studies are required to more fully understand how preexisting responses may influence vaccination or the immunological relatedness of the genotypes that could predict vaccine coverage or assess the potential benefit of additional VLPs. Previous analysis of clinical samples had found that MPL does not enhance the magnitude of immune responses to vaccine [33], and the work described here was meant to examine further if MPL influenced the breadth or character of the response to vaccine. No consistent differences were found in the strength or the breadth of responses when compared between the NOR-201 (MPL in vaccine) and NOR-210 (no MPL in vaccine) trials. Additionally, measuring IgG subclass levels for the GI.1 and GII.4 CN positive antibodies in these same samples did not show any changes in the ratios of the different IgG subclasses that may be expected if an adjuvant redirects the antibody response. These data indicate that addition of MPL is not required for effective vaccination in adults.

Levels of blocking antibodies have been identified as correlates of protection against norovirus disease [29,32]. We are unable to directly compare the blocking titers measured in this work against previous studies because of assay differences. However, as in the case with the HPV vaccine [43], the protective level of functional antibodies to nonvaccine VLPs may be considerably less than against vaccine antigens. Thus, even though the GMTs listed in Table 4 against the nonvaccine VLPs in the panel may be lower than to GI.1 and GII.4 CN, they could still be protective. Efficacy studies will be needed to define a true level of protection against nonvaccine genotypes.

One of the interesting results was how widespread the boosting was across all genotypes, suggesting that considerable cross-reactivity may be present. For the vaccine genotypes, the boosting was clear and consistent among individuals, especially across the GII.4 strains. Strong boosting of the GI.5 and GI.6 blocking antibodies also suggests a high degree of immunological relatedness with vaccine antigens. However, some individuals responded very strongly even when overall there was less than 4-fold rise and no consistent boosting among individuals. It will be of interest to identify why some individuals responded so well, and which epitopes they may be recognizing. Further dissection of these responses by depletion analyses and by evaluating monoclonal antibodies are being pursued to better define the epitopes that may be responsible for cross reactive blocking responses. Several blocking epitopes have been identified [45,46] and epitope-specific reagents may be better able to dissect the blocking antibody populations after vaccination, and possibly relate specific epitopes of most importance to protection against disease. Although cross-reactive epitopes may be present in norovirus particles, antibodies to these will not necessarily be expanded due to infection, or vaccination, since stronger type-specific antibodies can dominate the immune response. Repeated exposures to different variants of an antigen during chronic infection are believed to enhance cross-reactive responses, as suggested for the appearance of broadly neutralizing antibodies against HIV [47]. This sequential exposure to the various norovirus genotypes during childhood may lead to development of protective cross-reactive antibodies in adults [48]. Identifying these cross-reactive epitopes in adults could then lead to the design of antigens or vaccination strategies that would favor cross-reactivity and enhance the protective capability of vaccines.

## Figures and Tables

**Table 1:** Pig gastric mucin (PGM) binding by virus-like particles (VLPs) under different conditions of temperature (22 °C or 37 °C), buffer pH (6.5, 7.0 and 7.5), and skim milk percent (0% and 5%).

	Buffer pH 6.5				Buffer pH 7.0				Buffer pH 7.5			
	22 °C PGM		37 °C PGM		22 °C PGM		37 °C PGM		22 °C PGM		37 °C PGM	
	0%	5%	0%	5%	0%	5%	0%	5%	0%	5%	0%	5%
GI.1 (1968)	78 ^1^	38	100	61	79	32	96	49	83	38	97	57
GI.2 (2013)	59	42	100	57	49	38	77	47	54	41	81	50
GI.3 (2007)	68	60	95	91	72	48	96	81	72	58	100	81
GI.4 (2008)	83	20	100	22	76	13	95	14	75	21	93	25
GI.5 (2013)	81	45	100	33	63	37	95	38	47	32	43	35
GI.6 (2003)	91	75	71	42	100	66	44	22	99	89	18	8
GI.7 (2010)	74	28	100	33	38	29	53	31	80	35	98	41
GII.3 (2011)	68	60	95	91	72	48	96	81	72	58	100	81
GII.4 (CN)	79	55	100	83	76	56	79	76	79	63	33	62
GII.4 (2009)	76	59	98	72	77	61	100	74	74	66	97	86
GII.4 (2012)	68	60	95	91	72	48	96	81	72	58	100	81
GII.6 (2012)	77	53	100	65	79	50	98	55	70	59	92	68
GII.17 (1978)	80	67	100	89	66	62	83	75	74	69	75	74
GII.17 (2014)	85	53	96	68	86	57	95	72	89	64	100	83
GII.17 (2015)	87	66	98	90	81	55	98	86	80	57	100	88
GIV.1 (2012)	89	33	51	13	100	24	74	14	69	27	54	15

^1^ Values are relative percent binding with strongest measures assigned a value of 100%. Red shading represents the strongest binding and green the weakest binding.

**Table 2:** Titer comparisons from VLP blockade assays performed under different conditions of primary incubation (18 h and 1 h), PGM binding temperature (22 °C and 37 °C), and assay buffer pH (6.5 and 7.5).

	Primary Incubation	PGM Incubation	Assay Buffer pH	Final Conditions (All 18 h Primary)	
	18 h/1 h	37 °C/22 °C	pH 6.5/pH 7.5	Buffer pH	PGM
GI.1 (1968)	2.0	1.1	1.0	Both	37 °C
GI.2 (2013)	1.3	1.1	1.0	Both	37 °C
GI.3 (2007)	1.3	1.0	0.8	Both	37 °C
GI.4 (2008)	1.8	1.2	1.1	Both	37 °C
GI.5 (2013)	1.8	1.9	pH 7.5 Fail	pH 6.5	37 °C
GI.6 (2003)	1.1	37 °C Fail	pH 7.5 Fail	pH 6.5	22 °C
GI.7 (2010)	2.5	1.3	0.6	pH 7.5	37 °C
GII.3 (2011)	3.7	1.0	1.0	Both	37 °C
GII.4 (CN)	1.3	1.5	pH 7.5 Fail	pH 6.5	37 °C
GII.4 (2002)	1.1	1.1	0.9	pH 6.5	37 °C
GII.4 (2006a)	1.4	1.2	0.8	pH 6.5	37 °C
GII.4 (2006b)	1.2	1.0	pH 7.5 Fail	pH 6.5	37 °C
GII.4 (2009)	1.6	1.3	0.9	Both	37 °C
GII.4 (2012)	1.4	1.5	0.5	pH 7.5	37 °C
GII.6 (2012)	1.4	1.5	pH 7.5 Fail	pH 6.5	37 °C
GII.12 (2010)	1.1	2.2	pH 7.5 Fail	pH 6.5	37 °C
GII.17 (1978)	2.3	1.7	0.4	pH 7.5	37 °C
GII.17 (2014)	3.0	1.5	0.5	pH 7.5	37 °C
GII.17 (2015)	3.0	2.0	0.5	pH 7.5	37 °C
GIV.1 (2012)	1.0	37 °C Fail	pH 7.5 Fail	pH 6.5	22 °C

**Table 3:** Percentage of subjects with positive blockade titers before and after vaccination.

	Prevaccination % Positive			Postvaccination % Positive		
	NOR-201	NOR-210	Both	NOR-201	NOR-210	Both
GI.1 (1968)	32	29	31	88	92	90
GI.2 (2013)	32	46	39	52	67	59
GI.3 (2007)	52	50	51	56	54	55
GI.4 (2008)	28	29	29	40	42	41
GI.5 (2013)	56	54	55	96	79	88
GI.6 (2003)	36	46	41	84	88	86
GI.7 (2010)	36	38	37	64	67	65
GII.3 (2011)	64	63	63	72	79	76
GII.4 (CN)	92	67	80	100	100	100
GII.4 (2002)	92	88	90	100	100	100
GII.4 (2006a)	92	88	90	100	100	100
GII.4 (2006b)	88	71	80	100	96	98
GII.4 (2009)	60	58	62	96	88	92
GII.4 (2012)	84	63	73	100	96	98
GII.6 (2012)	84	79	82	84	96	90
GII.12 (2010)	72	50	61	72	75	73
GII.17 (1978)	88	92	90	96	100	98
GII.17 (2014)	60	58	59	84	75	80
GII.17 (2015)	44	50	47	76	71	73
GIV.1 (2012)	4	13	8	12	17	14

**Table 4:** Geometric mean titers before and after vaccination.

	Prevaccination GMTs			Postvaccination GMTs		
	NOR-201	NOR-210	Both	NOR-201	NOR-210	Both
GI.1 (1968)	23 (17–32)	28 (18–44)	25 (20–33)	308 (163–580)	397 (210–752)	349 (226–538)
GI.2 (2013)	25 (18–36)	39 (24–64)	31 (23–42)	59 (32–111)	101 (52–195)	77 (50–120)
GI.3 (2007)	44 (27–71)	48 (27–83)	46 (32–65)	58 (34–100)	76 (39–148)	66 (44–100)
GI.4 (2008)	25 (17–36)	33 (18–59)	29 (20–40)	57 (26–124)	62 (26–144)	59 (34–103)
GI.5 (2013)	56 (32–99)	70 (36–135)	62 (41–95)	509 (292–889)	417 (177–981)	462 (283–752)
GI.6 (2003)	27 (19–39)	42 (25–70)	34 (25–46)	194 (97–386)	207 (114–373)	200 (129–310)
GI.7 (2010)	35 (21–60)	42 (23–75)	38 (26–56)	91 (48–175)	117 (56–243)	103 (64–165)
GII.3 2011)	54 (33–88)	58 (36–94)	56 (40–78)	86 (49–150)	118 (68–204)	100 (69–147)
GII.4 (CN)	128 (85–192)	83 (47–148)	104 (74–146)	906 (605–1355)	1609 (1065–2433)	1200 (898–1604)
GII.4 2002)	313 (192–511)	225 (120–424)	266 (181–392)	2030 (1441–2860)	2896 (1673–5014)	2416 (1767–3304)
GII.4 (2006a)	428 (259–707)	317 (158–636)	370 (245–558)	2675 (1836–3897)	4079 (2296–7245)	3289 (2356–4591)
GII.4 (2006b)	121 (75–196)	99 (51–192)	110 (74–162)	711 (503–1006)	755 (436–1308)	732 (537–999)
GII.4 (2009)	64 (38–108)	64 (36–117)	64 (44–94)	256 (179–365)	378 (210–679)	310 (222–432)
GII.4 (2012)	110 (67–181)	62 (36–108)	83 (58–120)	566 (389–825)	543 (323–914)	555 (408–754)
GII.6 (2012)	124 (72–216)	94 (56–159)	108 (75–157)	178 (100–318)	176 (119–260)	177 (126–248)
GII.12 (2010)	62 (40–96)	55 (30–101)	58 (41–83)	89 (53–152)	98 (56–171)	93 (64–135)
GII.17 (1978)	150 (88–257)	134 (85–212)	142 (101–200)	361 (212–617)	368 (261–518)	364 (268–496)
GII.17 (2014)	52 (32–85)	50 (29–84)	51 (36–72)	103 (62–173)	80 (48–136)	91 (64–131)
GII.17 (2015)	37 (23–57)	35 (23–53)	36 (26–48)	76 (45–128)	68 (42–111)	72 (51–102)
GIV.1 (2012)	17 (13–21)	19 (14–25)	18 (15–21)	19 (14–24)	20 (15–28)	19 (16–24)

**Table 5:** Geometric mean fold-rise of titers after vaccination.

	NOR-201	NOR-210	Both
GI.1 (1968)	13.5 (7.8–23.3)	14.4 (7.6–27.5)	14.0 (9.3–20.9)
GI.2 (2013)	2.3 (1.5–3.8)	2.7 (1.6–4.5)	2.5 (1.8–3.5)
GI.3 (2007)	1.4 (1.1–1.9)	1.6 (1.2–2.2)	1.5 (1.3–1.9)
GI.4 (2008)	2.3 (1.3–3.9)	1.9 (1.3–2.9)	2.1 (1.5–2.9)
GI.5 (2013)	9.2 (5.0–17.2)	6.1 (3.3–11.2)	7.5 (4.9–11.5)
GI.6 (2003)	6.9 (3.8–12.6)	4.9 (2.9–8.2)	5.8 (4.0–8.6)
GI.7 (2010)	2.5 (1.6–4.0)	2.8 (1.8–4.5)	2.7 (1.9–3.7)
GII.3 (2011)	1.7 (1.3–2.3)	1.9 (1.2–3.2)	1.8 (1.4–2.4)
GII.4 (CN)	7.2 (4.4–11.9)	19.7 (10.3–37.5)	11.8 (7.8–17.9)
GII.4 (2002)	6.4 (3.9–10.6)	12.7 (7.5–21.4)	9.0 (6.2–12.9)
GII.4 (2006a)	6.0 (3.6–9.8)	13.1 (7.7–22.3)	8.7 (6.0–12.7)
GII.4 (2006b)	5.8 (3.3–10.0)	7.9 (4.7–13.3)	6.7 (4.7–9.7)
GII.4 (2009)	3.8 (2.3–6.1)	5.3 (2.9–9.8)	4.5 (3.1–6.5)
GII.4 (2012)	5.0 (2.8–8.8)	8.5 (5.2–14.0)	6.5 (4.5–9.4)
GII.6 (2012)	1.5 (1.2–1.9)	1.7 (1.2–2.4)	1.6 (1.3–2.0)
GII.12 (2010)	1.4 (1.1–1.7)	1.7 (1.2–2.3)	1.5 (1.3–1.9)
GII.17 (1978)	2.3 (1.6–3.3)	2.8 (1.9–4.0)	2.5 (2.0–3.3)
GII.17 (2014)	2.0 (1.4–2.7)	1.6 (1.1–2.2)	1.8 (1.4–2.2)
GII.17 (2015)	2.1 (1.5–2.9)	1.9 (1.3–2.6)	2.0 (1.6–2.5)
GIV.1 (2012)	1.1 (0.9–1.3)	1.1 (0.9–1.3)	1.1 (1.0–1.2)

## Appendix A

**Table A1:** Strain-specific information.

		VLP	Detection Antibody	
	Accession Number	Working Conc. (µg/mL)	Antigen	Working Conc. (µg/mL)
GI.1 (1968)	M87661	0.075	GI.1 (1968)	6.0
GI.2 (2013)	KF306212	0.3	GI.2 (2013)	6.0
GI.3 (2007)	FJ711164	0.1	GI.3 (2007)	6.0
GI.4 (2008)	GQ413970	0.1	GI.4 (2008)	6.0
GI.5 (2013)	KJ402295	0.15	GI.5 (2013)	6.0
GI.6 (2003)	KC998959	0.1	GI.6 (2003)	6.0
GI.7 (2010)	JN899243	0.1	GI.7 (2010)	6.0
GII.3 (2011)	KC597140	0.1	GII.3 (2011)	6.0
GII.4 (CN)	N/A	0.1	GII.4 (CN)	10
GII.4 (2002)	ABY27560	0.1	GII.4 (CN)	10
GII.4 (2006a)	ABL74391	0.075	GII.4 (CN)	10
GII.4 (2006b)	ABL74395	0.1	GII.4 (CN)	10
GII.4 (2009)	GU445325	0.1	GII.4 (CN)	10
GII.4 (2012)	JX459908	0.1	GII.4 (2012)	6.0
GII.6 (2012)	AB818400	0.15	GII.6 (2012)	6.0
GII.12 (2010)	KP064099	0.1	GII.12 (2010)	6.0
GII.17 (1978)	JN699043	0.15	GII.17 (1978)	6.0
GII.17 (2014)	LC043139	0.075	GII.17 (2014)	6.0
GII.17 (2015)	KP698931	0.075	GII.17 (2015)	6.0
GIV.1 (2012)	AFN61315	0.15	GIV.1 (2012)	6.0

**Table A2:** Blockade titers measured in sera from subjects enrolled in the NOR-201 clinical trial.

			GI.1	GI.2	GI.3	GI.4	GI.5	GI.6	GI.7	GII.3	GII.4	GII.4	GII.4	GII.4	GII.4	GII.4	GII.6	GII.12	GII.17	GII.17	GII.17	GIV.1
Study	Sub	Bleed	1968	2013	2007	2008	2013	2003	2010	2011	CN	2002	2006a	2006b	2009	2012	2012	2010	1978	2014	2015	2012
201	1	Pre	<30	<30	<30	<30	<30	<30	98	226	104	182	301	83	<30	105	142	<30	56	<30	<30	<30
201	1	Post	179	81	112	<30	659	73	1197	293	2698	3788	5318	1248	121	1131	250	<30	168	56	73	<30
201	2	Pre	46	<30	<30	178	122	66	72	101	134	334	543	106	254	130	93	42	174	128	82	<30
201	2	Post	2132	<30	<30	198	239	141	89	155	806	2025	2872	179	252	272	111	49	186	125	94	<30
201	3	Pre	<30	<30	44	<30	<30	69	<30	60	50	81	139	20	<30	<30	205	37	104	62	76	<30
201	3	Post	765	<30	110	<30	1076	2421	307	172	9267	9677	12578	4424	288	4934	874	115	1313	691	351	36
201	4	Pre	44	48	286	<30	67	<30	108	465	650	1540	1738	546	66	118	846	185	1123	484	182	<30
201	4	Post	2198	100	320	<30	155	<30	151	480	1020	2728	3727	756	184	211	962	191	1040	396	193	<30
201	5	Pre	<30	<30	<30	<30	<30	<30	<30	<30	<30	<30	<30	<30	<30	<30	<30	<30	58	<30	<30	<30
201	5	Post	238	<30	<30	<30	<30	<30	<30	<30	116	143	135	72	<30	72	<30	<30	404	127	93	<30
201	6	Pre	171	46	56	<30	237	60	290	57	91	241	352	55	49	32	185	617	319	181	100	<30
201	6	Post	94	151	97	228	1360	405	589	317	3491	5505	7292	403	74	601	522	769	693	241	168	<30
201	7	Pre	48	122	332	50	314	<30	386	74	293	564	829	491	142	549	196	132	126	<30	<30	<30
201	7	Post	1292	127	293	55	330	<30	349	113	847	2011	2766	1051	343	918	205	105	134	44	31	<30
201	8	Pre	<30	<30	<30	<30	<30	<30	<30	<30	73	242	324	56	<30	63	145	<30	123	<30	<30	<30
201	8	Post	199	<30	<30	<30	125	306	<30	<30	1160	2805	3864	929	258	1210	159	<30	191	79	37	<30
201	9	Pre	<30	<30	142	<30	<30	<30	<30	<30	101	277	392	91	95	104	<30	<30	<30	94	<30	<30
201	9	Post	135	<30	308	<30	147	<30	144	<30	910	3292	4081	1310	735	1421	<30	<30	204	95	<30	<30
201	10	Pre	<30	93	<30	122	81	157	<30	174	<30	<30	<30	<30	179	<30	644	87	599	300	179	208
201	10	Post	430	622	<30	1063	3181	2550	234	263	108	652	617	881	128	127	1602	375	2563	1273	734	199
201	11	Pre	<30	<30	<30	37	<30	<30	<30	<30	36	130	183	<30	<30	<30	<30	<30	<30	45	<30	<30
201	11	Post	224	<30	<30	655	703	142	<30	66	1302	3195	4261	451	131	415	<30	<30	72	79	<30	<30
201	12	Pre	<30	<30	<30	81	34	<30	<30	88	362	801	1134	217	107	296	301	136	183	85	53	<30
201	12	Post	789	135	<30	3591	4683	1039	204	146	666	1290	1671	392	184	323	343	164	199	83	75	<30
201	13	Pre	<30	70	<30	<30	<30	<30	<30	<30	359	1031	1311	382	173	350	<30	<30	<30	<30	<30	<30
201	13	Post	362	554	57	<30	144	34	<30	<30	623	1770	2352	805	381	519	<30	<30	<30	<30	<30	<30
201	14	Pre	125	<30	196	<30	88	<30	<30	30	397	1060	1406	660	301	492	88	114	42	<30	<30	<30
201	14	Post	2605	<30	205	58	161	44	<30	102	597	1426	2019	951	506	684	88	165	61	<30	<30	<30
201	15	Pre	<30	46	40	<30	537	<30	67	457	306	1130	1190	268	IND	397	3224	215	933	210	362	<30
201	15	Post	186	459	422	318	3664	1755	733	62	641	1349	1484	767	356	925	307	333	1100	134	142	<30
201	16	Pre	<30	<30	<30	<30	68	<30	<30	165	236	658	895	146	155	244	177	42	610	<30	<30	<30
201	16	Post	163	<30	<30	<30	673	420	66	190	636	1577	2004	385	281	367	217	147	1193	61	53	<30
201	17	Pre	43	<30	106	<30	234	<30	<30	<30	47	124	188	66	<30	32	54	67	143	<30	<30	<30
201	17	Post	2395	<30	135	<30	367	80	<30	<30	329	851	1180	607	351	444	106	84	285	38	61	<30
201	18	Pre	<30	<30	<30	<30	<30	49	<30	<30	106	473	697	166	134	62	146	74	102	97	46	<30
201	18	Post	437	<30	<30	<30	502	193	<30	<30	636	1653	2283	485	406	242	214	117	586	138	100	<30
201	19	Pre	49	165	233	<30	830	181	499	78	112	354	486	226	169	156	310	127	1448	149	168	<30
201	19	Post	2922	146	211	<30	869	225	479	380	2351	5424	7353	1820	833	1980	630	287	2306	423	649	<30
201	20	Pre	<30	<30	145	104	<30	<30	<30	<30	102	371	523	63	<30	90	51	103	87	<30	<30	<30
201	20	Post	264	1100	226	2561	1699	118	84	<30	715	1734	2656	326	161	340	69	110	118	<30	<30	<30
201	21	Pre	38	<30	<30	176	370	44	61	35	204	629	925	257	130	277	156	94	107	37	<30	<30
201	21	Post	183	150	<30	608	4540	813	675	118	682	1976	2638	1121	576	921	953	189	360	137	92	<30
201	22	Pre	<30	93	149	<30	52	120	471	150	91	205	294	102	<30	104	186	124	289	133	80	<30
201	22	Post	296	503	181	<30	891	971	583	1433	3555	4572	6781	3082	722	1336	717	635	3631	568	839	99
201	23	Pre	<30	<30	83	<30	<30	<30	<30	<30	305	807	1053	474	391	367	35	<30	107	36	<30	<30
201	23	Post	<30	<30	<30	<30	138	1170	<30	31	1939	4237	6642	744	509	507	104	<30	227	51	40	<30
201	24	Pre	<30	<30	72	<30	293	<30	<30	60	168	336	489	194	<30	147	65	133	193	<30	<30	<30
201	24	Post	<30	44	84	<30	471	72	50	66	627	1466	2336	547	150	336	83	189	243	<30	<30	<30
201	25	Pre	<30	<30	<30	<30	<30	71	<30	264	336	705	958	341	303	596	469	119	777	308	163	<30
201	25	Post	<30	<30	<30	<30	338	858	<30	254	767	1542	1728	832	382	986	508	115	977	388	216	<30

Blockade titers below the assigned lower limit of quantitation of 30 were given a value of <30. IND represents indeterminant titers in which sample titer could not be calculated for both the initial and repeat testing.

**Table A3:** Blockade titers measured in sera from subjects enrolled in the NOR-210 clinical trial.

			GI.1	GI.2	GI.3	GI.4	GI.5	GI.6	GI.7	GII.3	GII.4	GII.4	GII.4	GII.4	GII.4	GII.4	GII.6	GII.12	GII.17	GII.17	GII.17	GIV.1
Study	Sub	Bleed	1968	2013	2007	2008	2013	2003	2010	2011	CN	2002	2006a	2006b	2009	2012	2012	2010	1978	2014	2015	2012
210	26	Pre	<30	179	98	<30	280	<30	259	118	297	889	1467	1077	187	350	1440	518	1154	408	125	<30
210	26	Post	310	585	226	79	2654	397	1875	<301	827	1823	2632	1696	230	811	1251	493	1378	458	147	<30
210	27	Pre	<30	<30	342	<30	372	<30	198	217	<30	80	100	<30	<30	<30	62	143	103	34	<30	<30
210	27	Post	77	<30	514	<30	1090	213	367	351	1748	2625	3443	548	198	193	132	203	188	68	52	<30
210	28	Pre	85	86	53	65	166	<30	122	64	<30	74	101	<30	52	<30	139	<30	101	106	32	<30
210	28	Post	612	352	<30	699	962	88	185	58	1526	4310	5514	538	309	375	253	<30	303	123	89	78
210	29	Pre	690	540	444	1577	864	593	719	276	79	448	619	520	498	180	359	<30	164	682	429	264
210	29	Post	1464	874	1265	2105	3183	1684	3111	408	10666	15312	20102	997	779	789	376	67	459	576	350	246
210	30	Pre	<30	<30	<30	<30	<30	<30	<30	<30	364	928	1282	427	194	360	<30	<30	102	<30	<30	30
210	30	Post	199	<30	<30	<30	<30	<30	<30	<30	2240	4379	5835	2548	926	1896	80	38	206	<30	<30	65
210	31	Pre	<30	98	66	166	81	411	<30	<30	<30	<30	<30	<30	<30	<30	<30	<30	134	54	50	<30
210	31	Post	525	419	161	1311	2247	1384	112	1091	275	104	118	129	<30	303	369	211	1529	530	468	<30
210	32	Pre	<30	<30	<30	<30	295	<30	105	<30	<30	100	129	49	<30	32	95	<30	118	<30	31	<30
210	32	Post	3440	<30	<30	<30	522	39	171	200	3707	6667	8305	3587	1217	2735	134	51	342	93	116	<30
210	33	Pre	<30	<30	70	<30	<30	<30	129	152	335	577	950	173	34	56	40	<30	35	<30	<30	<30
210	33	Post	392	157	253	<30	1671	601	690	181	2578	4770	6373	1039	678	1066	93	<30	318	<30	69	<30
210	34	Pre	<30	<30	90	504	<30	119	<30	88	<30	<30	<30	<30	<30	<30	34	34	148	<30	47	<30
210	34	Post	311	1433	165	4994	1643	579	213	413	96	81	92	95	<30	122	58	194	386	84	190	<30
210	35	Pre	<30	<30	<30	<30	<30	90	<30	<30	74	159	227	90	48	84	50	<30	54	<30	<30	<30
210	35	Post	176	<30	<30	<30	203	563	<30	41	557	1106	1616	668	340	581	74	<30	171	<30	<30	<30
210	36	Pre	<30	<30	<30	<30	<30	260	<30	<30	405	1142	1972	112	89	65	206	121	66	<30	<30	<30
210	36	Post	203	<30	<30	<30	90	1031	<30	<30	2209	6022	7565	1322	752	1114	483	259	1387	186	174	<30
210	37	Pre	125	58	314	<30	536	76	<30	100	103	241	356	75	310	71	130	167	148	32	50	<30
210	37	Post	4379	142	370	89	1145	141	44	123	6345	14297	24490	2048	1067	1138	164	267	235	39	60	<30
210	38	Pre	88	104	73	50	423	<30	<30	234	137	400	460	330	152	97	268	100	497	117	55	<30
210	38	Post	278	455	300	199	4583	346	498	439	777	2090	3547	999	362	329	385	169	791	139	72	<30
210	39	Pre	<30	<30	<30	<30	<30	101	<30	<30	<30	59	72	<30	<30	<30	<30	<30	<30	<30	<30	<30
210	39	Post	<30	<30	<30	<30	<30	212	<30	<30	2283	5403	6935	333	200	332	51	82	110	<30	<30	<30
210	40	Pre	<30	56	<30	<30	<30	<30	<30	174	230	1328	2517	459	311	110	449	<30	661	<30	<30	<30
210	40	Post	2381	94	<30	<30	212	<30	<30	218	2427	6321	9443	693	465	303	481	43	724	37	37	<30
210	41	Pre	<30	<30	168	<30	<30	61	149	211	<30	33	71	<30	<30	<30	264	222	411	103	<30	<30
210	41	Post	<30	<30	294	<30	132	119	247	246	3317	6645	9385	1157	473	388	396	307	521	168	90	<30
210	42	Pre	<30	<30	<30	<30	70	<30	<30	<30	920	1495	3847	1820	738	597	<30	<30	<30	37	<30	<30
210	42	Post	350	668	173	<30	1699	383	169	<30	1549	6600	7032	3434	1618	1361	124	<30	231	36	<30	<30
210	43	Pre	<30	<30	<30	<30	103	<30	<30	86	241	458	556	425	218	315	154	219	320	139	86	<30
210	43	Post	402	181	117	<30	3156	720	296	104	1235	2287	4880	1922	833	1244	196	317	613	163	119	<30
210	44	Pre	<30	<30	<30	182	190	<30	<30	58	86	386	528	31	<30	<30	110	241	79	<30	<30	<30
210	44	Post	768	82	<30	2951	3796	432	98	67	3682	4034	11418	1292	1071	1436	131	283	144	<30	<30	<30
210	45	Pre	<30	<30	<30	<30	<30	91	<30	265	274	1270	2231	518	195	425	195	602	546	195	176	<30
210	45	Post	123	<30	<30	<30	<30	208	<30	296	1532	7547	7177	1469	365	1377	277	644	953	342	377	<30
210	46	Pre	90	61	<30	<30	<30	<30	<30	<30	141	141	211	36	<30	<30	<30	<30	112	<30	<30	<30
210	46	Post	1660	68	<30	<30	<30	<30	<30	<30	786	1297	2483	99	<30	46	<30	<30	179	<30	<30	<30
210	47	Pre	<30	210	<30	<30	<30	<30	<30	<30	<30	<30	<30	<30	<30	<30	157	<30	82	174	<30	<30
210	47	Post	763	367	<30	<30	<30	34	<30	476	1794	663	663	<30	659	<30	160	<30	75	112	<30	<30
210	48	Pre	226	304	284	293	262	107	274	89	97	346	334	208	168	117	153	481	108	245	130	86
210	48	Post	147	129	403	157	521	235	641	263	2405	4927	8090	1335	1047	1484	265	478	420	317	207	61
210	49	Pre	37	71	435	<30	725	135	634	94	231	895	1325	200	IND	190	152	259	310	106	101	<30
210	49	Post	4327	154	550	49	1209	323	889	109	1681	3720	5461	1684	1624	1398	176	268	389	117	132	<30

Blockade titers below the assigned lower limit of quantitation of 30 were given a value of <30. IND represents indeterminant titers in which sample titer could not be calculated for both the initial and repeat testing.
